# Genetic diversity of *Actinobacillus lignieresii *isolates from different hosts

**DOI:** 10.1186/1751-0147-53-6

**Published:** 2011-02-08

**Authors:** Branko Kokotovic, Øystein Angen, Magne Bisgaard

**Affiliations:** 1 Division of Veterinary Diagnostics and Research, National Veterinary Institute, Technical University of Denmark, Bülowsvej 27, DK-1790 Copenhagen V, Denmark; 2 Department of Veterinary Disease Biology, Faculty of Life Sciences, University of Copenhagen, Stigbøjlen 4, DK-1870 Frederiksberg C, Denmark

## Abstract

Genetic diversity detected by analysis of amplified fragment length polymorphisms (AFLPs) of 54 *Actinobacilus lignieresii *isolates from different hosts and geographic localities is described. On the basis of variances in AFLP profiles, the strains were grouped in two major clusters; one comprising strains isolated from horses and infected wounds of humans bitten by horses and another consisting of strains isolated from bovine and ovine hosts. The present data indicate a comparatively higher degree of genetic diversity among strains isolated from equine hosts and confirm the existence of a separate genomospecies for *A. lignieresi*-like isolates from horses. Among the isolates from bovine and ovine hosts some clonal lines appear to be genetically stable over time and could be detected at very distant geographic localities. Although all ovine strains investigated grouped in a single cluster, the existence of distinct genetic lineages that have evolved specificity for ovine hosts is not obvious and needs to be confirmed in other studies.

## Findings

*Actinobacillus lignieresii *is a commensal of the oropharynx and rumen in cattle and sheep and has also been found in the oral cavity of healthy horses [1]. In bovine and ovine hosts the organism may cause pyogranulomatous inflammation, especially of the upper alimentary tract [2]. In horses it has been found in association with pyemic processes of soft tissues [3,4] and cases of stomatitis [1]. The virulence factors of *A. lignieresii *remain unknown. Different strains may vary in their ability to induce disease [5], but little progress has been made to identify reliable markers that will allow their epidemiological tracing. In addition, there is also limited information concerning genetic diversity in the natural population of the species.

Within the family *Pasteurellaceae *Pohl 1981 most taxa seem to be host specific [1]. However, very little is known about factors governing the ecological preferences that these taxa show for specific mucosal surfaces and hosts. *Actinobacillus pleuropneumoniae *and *A. lignieresii *are phenotypically very similar and their 16S rDNA sequences differ only by two nucleotides [6]. Whole genome fingerprinting by amplified fragment length polymorphism (AFLP) analysis, however, provided a clear separation of these taxa affecting pigs and ruminants, respectively [7]. Distinct genetic lineages within the taxon 2 and 3 complex of Bisgaard also seem to have evolved host specificity for *Columbidae, Anatidae *and *Psittacidae*, respectively [8]. Bojesen *et al. *[9] suggested the divergence of at least three distinct *Mannheimia granulomatis *lineages that may have adapted to cervine, bovine and leporine hosts, respectively. In addition, Bojesen *et al. *[10] also suggested the existence of host adapted lineages within *M. varigena*. AFLP analysis has also separated bovine and ovine isolates of *Bibersteinia trehalosi *indicating the existence of separate ecotypes [11]. Evidence as to the existence of clonal lineages of *Gallibacterium anatis *adapted to different sites within the same animal has also been demonstrated by AFLP [12].

The aims of the present study were to investigate *A. lignieresii *isolates from different hosts in order to (1) determine discriminatory potential of AFLP for subspecies differentiation, (2) examine intraspecies genetic diversity and (3) investigate whether bovine and ovine isolates of *A. lignieresii *represent host specific subclones of this taxon.

The test population used in the study consisted of 54 *A. lignieresii *strains isolated from bovine (n = 37), equine (n = 7) and ovine (n = 6) hosts, as well as one strain without host information and three strains isolated from infected wounds of humans bitten by horses (Table 1). The tested strains originated from different geographic localities including Australia, Belgium, Denmark, Norway, Sweden, United Kingdom, USA and Zimbabwe (Table 1). All strains were grown on Columbia agar (Oxoid A/S, Greve, Denmark) supplemented with 5% bovine blood and harvested after 48-hours incubation at 37°C in atmospheric air.

**Table 1:** *Actinobacillus lignieresii *strains analysed

Strain designation	Source	Host	Country
CCUG 18727	Abscess	Bovine	Australia
Y4927	Granuloma	Bovine	Australia
CCUG 18728	Lymph node	Bovine	Australia
Z4479-1	Lymph node	Bovine	Australia
93303-45	Joint	Bovine	Belgium
B96/11	unknown	Bovine	Belgium
C1005	Granuloma	Bovine	Denmark
C1017	Granuloma	Bovine	Denmark
C1020	Granuloma	Bovine	Denmark
C1021-1	Granuloma	Bovine	Denmark
C1021-2	Granuloma	Bovine	Denmark
C1024	Granuloma	Bovine	Denmark
C1033	Granuloma	Bovine	Denmark
C1080-2	Granuloma	Bovine	Denmark
C1129-1	Granuloma	Bovine	Denmark
C1129-2	Granuloma	Bovine	Denmark
C1130-1	Granuloma	Bovine	Denmark
C1215-1	Granuloma	Bovine	Denmark
C1231-1	Granuloma	Bovine	Denmark
C1245-2	Granuloma	Bovine	Denmark
C1245-3	Granuloma	Bovine	Denmark
C1508-1	Granuloma	Bovine	Denmark
C1687	Granuloma	Bovine	Denmark
C772	Granuloma	Bovine	Denmark
C823	Granuloma	Bovine	Denmark
C867	Granuloma	Bovine	Denmark
C872	Granuloma	Bovine	Denmark
CCUG 27361	Leg abscess	Bovine	Sweden
CCUG 27360	Lung	Bovine	Sweden
541/73	Granuloma	Bovine	UK
CCUG 22228	Lymph node	Bovine	UK
NCTC 4985	unknown	Bovine	UK
CCUG 23133	unknown	Bovine	unknown
NCTC 4191	Glands	Bovine	USA
ATCC 49236	unknown	Bovine	USA
AC3	Granuloma	Bovine	Zimbabwe
AC4	unknown	Bovine	Zimbabwe
F126	Oral cavity	Equine	Denmark
F127	Oral cavity	Equine	Denmark
F128	Oral cavity	Equine	Denmark
F258	Oral cavity	Equine	Denmark
F264	Oral cavity	Equine	Denmark
C5309-b	Stomatitis	Equine	Denmark
F429	Stomatitis	Equine	Denmark
T354/87	Wound *	Human	Australia
P1293	Wound *	Human	Denmark
F414	Wound *	Human	Norway
CCUG 38958	Abscess	Ovine	Sweden
A7	Abscess	Ovine	UK
A3	Lung	Ovine	UK
A6	Lymph node	Ovine	UK
HPA 107	Rumen	Ovine	UK
HPA 119	Rumen	Ovine	UK
Smith 40	unknown	unknown	Australia

* due to horse bite.

Bacterial genomic DNA was extracted by using EasyDNA^® ^kit (Invitrogen A/S, Taastrup, Denmark) according to the manufacturer's instruction. AFLP reaction was performed by using *Eco*RI and *Bsp*DI restriction enzymes as described previously [7]. Amplification products were detected on an ABI377 automated sequencer (Applied Biosystems, Naerum, Denmark) according to the manufacturer's instructions. Data collection and pre-processing were done by using GeneScan 3.1 software (Applied Biosystems). Numerical analysis was performed using BioNumerics software (Applied Maths, Sint-Martens-Latem, Belgium). In order to assess the reproducibility of the AFLP profiles, DNA specimens of seven strains were analysed in triplicates. Reproducibility was determined by direct comparison of densiometric curves by using Pearson product-moment correlation coefficient. Levels of similarity between normalized fingerprints were calculated by using binary Dice similarity coefficient (*S*_D_). Clustering of fingerprints was performed with the unweighted pair group method using arithmetic averages (UPGMA). Significance of the clusters was determined by bootstrap analysis with 1000 samplings. The discriminatory power of AFLP was calculated by using Simpson's index of diversity [13].

Banding patterns obtained by amplification of *Eco*RI-*Bsp*DI DNA templates with AFLP primers consisted of approximately 60 and 90 fragments in the size range 50-500 bp for equine/human and bovine/ovine isolates, respectively. The repeated analysis of identical samples showed highly reproducible result, although some variance in fluorescence intensity of AFLP fragments having identical size was observed. The variability observed was not sufficient to alter conclusions concerning relationship of one strain to another. Numerical comparison of AFLP densiometric curves of seven identical samples tested in triplicates showed an overall similarity level of 97.6 ± 1.8%.

In the present study 54 strains of *A. lignieresii *of animal and human origin were examined for genomic variation by using AFLP analysis. Among the strains tested 43 unique AFLP profiles were detected. Discriminatory index of the AFLP assay was 0.99.

**Figure 1:**
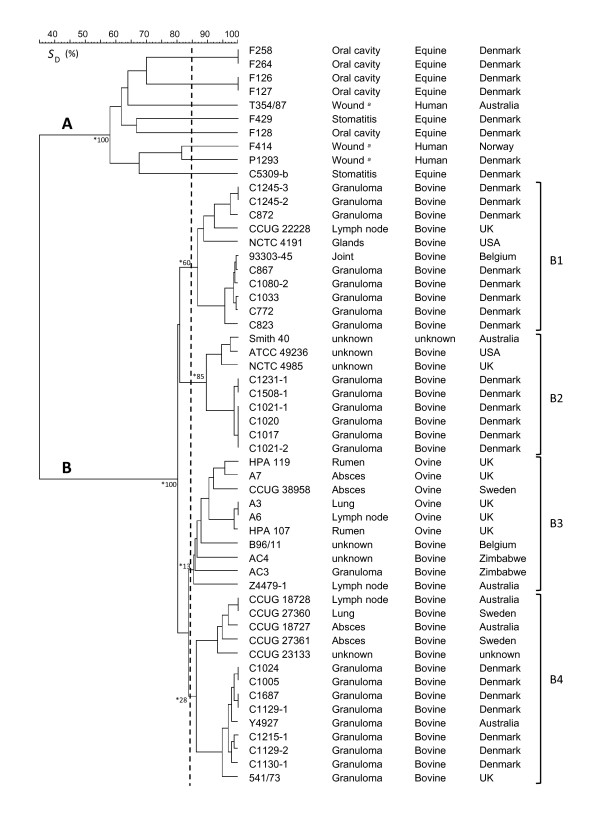
**Relatedness between *Actinobacillus lignieresii *isolates**. Similarity of AFLP profiles was calculated by using Dice similarity coefficient (*S*_D_) and the dendrogram was produced by using UPGMA. A and B, two major clusters detected by analysis of AFLP data; B1-B4, four subclusters detected among the bovine/ovine strains; * Values indicating significance of specific nodes obtained by bootstrap analysis. *^a ^*isolates from wounds due to horse bite.

Numerical analysis of AFLP data revealed two clearly distinguishable clusters; one consisting solely of strains isolated from horses and humans (cluster A; Figure 1), the other comprising strains of bovine and ovine origin (cluster B, Figure 1). Overall similarity between these two clusters was only 35%.

The 10 analysed equine/human isolates showed eight distinct AFLP patterns forming a cluster at the linkage level of 58% (Figure 1; cluster A). With the exception of the strains F258 and F264 which had identical AFLP profiles, as well as strain F126, which had an AFLP profile identical to F127, all other strains within the group showed widely different genomic fingerprints (data not shown). That is particularly interesting in the light of the fact that the majority of equine/human strains tested derived from a relatively small geographic area, i.e. Denmark. The substantial chromosomal variability detected may indicate that changes in the genetic makeup within the group occur at a relatively high frequency. Isolates from horses and infected wounds of humans bitten by horses have previously been examined for their phenotypic characteristic, sequence variation in 16S rRNA genes and DNA-DNA hybridization by Christensen *et al. *[14]. Results obtained showed that these isolates represent a unique group of organisms, which is genetically, but not phenotypically, distinct from the type strain of *A. lignieresii*. Whole genome fingerprinting by AFLP analysis conducted in the present study provide further evidence to support the inclusion of these isolates in a distinct genomospecies of *Actinobacillus *as proposed by Christensen *et al. *[14].

The 44 analysed strains of bovine/ovine origin showed 35 distinct AFLP profiles forming a cluster at the linkage level of 80% (Figure 1; cluster B). Within the cluster, four subgroups could be recognised at the arbitrarily chosen cut-off value of 85% similarity (Figure 1; B1-B4). All of the subgroups detected within the bovine/ovine cluster consisted of strains derived from different geographic regions. Interestingly, within the cluster, many strains showed highly similar AFLP profiles in spite of widely different time and locality of isolation. For example, AFLP profile of strain 93303-45, which was isolated from a bovine joint in Belgium, differed by a single fragment from the profile obtained from strain C867 which was isolated from a bovine granuloma in Denmark. Moreover, strain CCUG 18728, which was isolated from a bovine lymph node in 1985 in Australia, showed an AFLP fingerprint identical to that obtained from strain CCUG 27360, which was isolated from a bovine lung in 1990 in Sweden. Under the assumption that identical AFLP profiles among different bacterial strains signify a direct descent from a common progenitor, results obtained in the present study indicate the existence of stable clonal lines in the natural population of *A. lignieresii *from bovine/ovine hosts.

One of the objectives of the present study was to investigate whether bovine and ovine isolates represent host specific genetic lineages of *A. lignieresii*. As revealed by numerical analysis of AFLP data, all ovine strains were included in a single subgroup (Figure 1; subgroup B3), showing relatively high (90%) overall similarity of their AFLP profiles. However, the subgroup B3 included also bovine strains from Australia, Belgium and Zimbabwe, making it difficult to draw a firm conclusion concerning the existence of clonal lines specific for ovine hosts. In that connection it is worth noting that, according to the bootstrap values, the subgroup B3 appears to be the least stable of all recognized subgroups in the bovine/ovine clusters, and is therefore likely to be re-shuffled by inclusion of additional strains.

In conclusion, analysis of *Eco*RI-*Bsp*DI AFLP markers has been shown to represent a sensitive and reliable approach for differentiation of *A. lignieresii*, which render it useful for both classification and epidemiological tracing of individual clones. The results of the present study revealed a substantial degree of genetic diversity among the strains isolated from horses and infected wounds of humans bitten by horses, while a comparatively lower degree of genetic diversity was observed for strains of bovine/ovine hosts. Some clonal lines among bovine/ovine strains appear to be genetically stable over the time and can be found in very distant geographic locations. However, the existence of clonal lines that are adapted specifically to ovine hosts is not obvious and further studies are needed for full clarification.

## Competing interests

The authors declare that they have no competing interests.

## Authors' contributions

BK participated in the design of the study, carried out AFLP analysis and drafted the manuscript.

ØA participated in the design of the study and carried out bacteriological examination of the isolates.

MB participated in the design of the study, carried isolation and bacteriological examination of the isolates and drafted the manuscript.

All authors read and approved the final manuscript.
